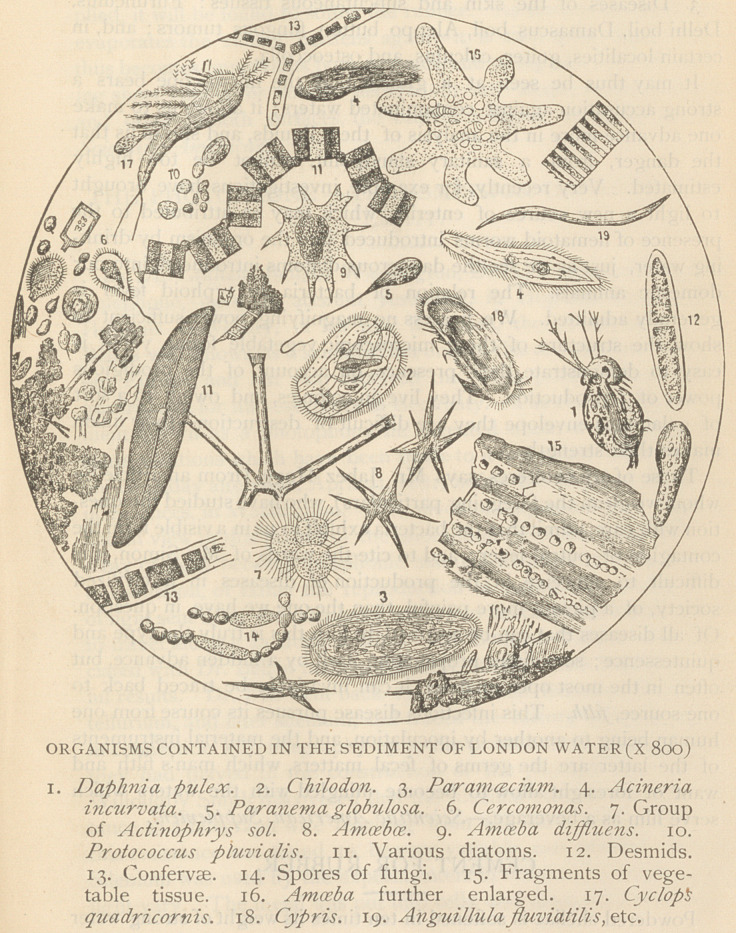# Microscopical Analysis of Water

**Published:** 1881-04

**Authors:** 


### THE MICROSCOPICAL ANALYSIS OF WATER.

Chemical analysis is powerless to reach those delicate particles which constitute organic germs. There exists no method of getting at the actual weight of these impurities, and it is impossible for the chemist to say what is the proportion of these miasms that may do injury to the health. Very foul water may sometimes be drunk for a long while without causing any apparent harm, and all at once accidents may supervene which may demand attention. The malady which attacks those who make use of such water, while it spares the neighbors employing water from another source, is a contageous one. An inquest being opened, demonstrates either that the dejections of a person affected with an infectious disease have infiltered into the water, or else that the source, already impure, has undergone an alteration of temperature sufficient to bring about rapid putrefaction. There are few physicians who have not had to authenticate cases of this nature. The sixth report of the London Commission on Insalubrious Waters, and statistical report of July 20, 1879, offer numerous examples of them. Facts demonstrate that it is not the organic matter of itself that renders water unhealthy, but it is the organized bodies or germs which have entered the liquid or which have developed very abundantly therein under favorable conditions.

Prof. Frankland has established it as a rule that we may admit, that all water containing 1.5 of organic nitrogen in 20,000 is a really dangerous one, and that the smaller the quantity of nitrogen in proportion to the organic carbon, the less chance there is of meeting animal or vegetable impurities. It must be remarked, however, that it is impossible to mark out a very precise limit in so delicate a question. The data upon which an analysis of water rests will serve, then, only as aids in forming a judgment, which will be modified by circumstances, as to the source, the hour, the locality from which the water has been taken, etc. It may turn out that it has been collected precisely over the point where the infectious matters are entering the river and changing the character of its waters.

The germs of specific diseases have hitherto escaped the ablest chemical analysis. Waters which are apparently clear and limpid may contain in solution or in suspension minute quantities of organic matters and become turbid on the application of heat. Matters that are most quickly oxidizable are the most dangerous ones. No known process of filtration will render infected water inoffensive or sufficiently pure to serve for potable use. The most palpable forms of impurities which are met with in river water escape the observation of the chemist because the reagents employed by him destroy these embryonic forms of animal and vegetable life. For these various reasons, the most efficacious examination is a physical analysis, and the microscope becomes a valuable aid to the test tube.

When we wish to submit water to a microscopic examination, it is necessary to take a sufficiently large bottle of the liquid to be tested, and to expose it for a day or two to a moderate heat in a well-lighted room, when there will form a deposit covering the bottom of the vessel. This deposit is carefully removed by the aid of a pipette or glass tube, long enough to reach the bottom of the vessel without producing too much agitation. Then this sediment is placed on a glass slide for examination under a 0.2 inch objective. If the water has been rendered foul by organic infiltrations, there will be observed therein a great number of minute corpuscles—epithelium, muscular fibres, starch granules, hairs, ligneous fibres, fungi, confervæ, diatoms, and, occasionally, infusoria (like the *Paramœcium*) or entomostraca. The existence of these different kinds of infusoria in water does not always indicate extreme danger, but the abundance of them generally proves, especially in summer, that there is a certain amount of organic impurities in the liquid of a suspicious nature. In reality, unless these beings find sufficient nourishment they promptly die, and their accumulated *debris* furnish a new quantity of putrescible matter which remains in suspension and easily passes through all filters. The annexed engraving represents the organisms observed with the microscope in the sediment deposited by the water supplied to London at the southern part of the Thames. We may count therein no less than nineteen different genera.

The most practical means of arriving at the real state of the question—the importance of these organisms from a sanitary standpoint, and their probable effect on human beings when they are introduced into the economy—is to enumerate the different diseases to which they give rise.

1. Diseases of the digestive apparatus; Dyspepsia, diarrhea, cholerine, and dysentery, to which should be added entozoa or internal parasites—tenias, ascarides, filarias, and diatoms.

ORGANISMS CONTAINED IN THE SEDIMENT OF LONDON WATER (X 800)

1. *Daphnia pulex.* 2. *Chilodon.* 3. *Paramœcium.* 4. *Acineria incurvata.* 5. *Paranema globulosa.* 6. *Cercomonas.* 7. Group of *Actinophrys sol.* 8. *Amœbæ.* 9. *Amœba diffluens.* 10. *Protococcus pluvialis.* 11. Various diatoms. 12. Desmids. 13. Confervæ. 14. Spores of fungi. 15. Fragments of vegetable tissue. 16. *Amœba* further enlarged. 17. *Cyclops quadricornis.* 18. *Cypris.* 19. *Anguillula fluviatilis,* etc.

2. Specific diseases: Malarial fevers, typhoid or putrid fever, cholera, yellow fever, and relapsing fever.

3. Diseases of the skin and subcutaneous tissues: Furunculus, Delhi boil, Damascus boil, Aleppo button, fungous tumors; and, in certain localities, goiter, calculus, and osteoccolla.

It may thus be seen at a glance that the microscope bears a strong accusation against contaminated waters; it allows us to make one advance more in the analysis of these liquids, and shows us that the danger, from a sanitary standpoint, cannot be too highly estimated. Very recently, for example, investigations have brought to light a new source of enteritis, which may be attributed to the presence of nematoid worms introduced into the organism by drinking water, just as we see the dangerous diatoms introduced into our domestic animals. The relation of bacteria to typhoid fever is generally admitted. We possess no magnifying power sufficient to show the structure of these microscopic vegetable forms, yet it is easy to demonstrate their presence, on account of the prodigious power of reproduction. They live in colonies, and owing to a sort of gelatinous envelope they are difficult of destruction—their union makes their strength.

Those of my confreres, says Mr. Jabez Hogg (from an article by whom we glean the foregoing particulars), who have studied the question with care, admit that the bacteria exhibit to us in a visible form the contagion of putrid fever; and to cite the words of Mr. Simon, it is difficult to conceive, in the production of diseases in a civilized society, of a picture more painful than the one we have in question. Of all diseases that can be ascribed to filth this is truly the type and quintessence; sometimes propagating itself by a hidden advance, but often in the most open manner, it can invariable be traced back to one source, *filth.* This infectious disease pursues its course from one human being to another by inoculation, and the material instruments of the latter are the germs of fecal matters, which man's filth and want of foresight allow to become mingled with the waters which serve him as a beverage.—*Scientific American Supplement.*

## Figures and Tables

**Figure f1:**